# Supplementary material for: Administration of Momordica charantia Enhances the Neuroprotection and Reduces the Side Effects of LiCl in the Treatment of Alzheimer’s Disease
Source: Nutrients. 2018 Dec 3;10(12):1888. doi: 10.3390/nu10121888 (PMC6316175; doi:10.3390/nu10121888)
Supplement: Supplementary file 1 [file nutrients-10-01888-s001.pdf]

Supplemental Materials and Methods

Primary Mouse Hippocampal Neuronal Cultures and Drug Treatments

The method used to prepare primary cell cultures was the same as that previously described [1]. Pregnant C57BL/6J mice (National Laboratory Animal Center; NLAC; Taipei, Taiwan) were sacrificed, and the hippocampi were dissected from the brains of day 16–18 embryos. Tissues were trypsinized (0.05%) for 15 min at 37 °C. The cells were plated on 24-well plates (3×10^4 cells/well) that were pre-coated with 100 μg/mL poly-L-lysine and were then cultured in Neurobasal media (GIBCO, Carlsbad, CA, USA) supplemented with 2% B27 (GIBCO), 0.5 mM glutamine (GIBCO), 25 μM glutamate (Sigma-Aldrich), penicillin/streptomycin (GIBCO, 20 unit/mL), 1 mM HEPES (Sigma-Aldrich), and 1% heat-inactivated donor horse serum (GIBCO). On days in vitro (DIV) 4 and 7, cytosine arabinoside (2 μM, Sigma-Aldrich) was added to reduce the glial cell population. On DIV 9, the cells were treated with 4 different MC varieties (Nos. 2, 3, 5, and 5523) at 1, 10, 50 and 100 μg/mL, LiCl at 2.5, 5, 10 and 50 mM (Sigma-Aldrich), and 10 mM glucose for 24 h (Figure. S1). The neuroprotective effects of LiCl plus with MC5 or 5523 were further examined in mouse primary hippocampal neurons treated with 10 nM wortmannin (WT)/GF-109203X (GFX) or DMSO on DIV 9 for 1 h (Figure. S2). All samples were harvested for the immunocytochemistry analysis and lactate dehydrogenase (LDH) assay. The preparation of mouse primary hippocampal neuronal cultures was approved by the Institutional Animal Care and Use Committee (IACUC) of National Taiwan Normal University, Taipei, Taiwan (Permit Number: 103004). The animals were anesthetized with avertin (0.4 g/kg of body weight) and decapitated; all efforts were made to minimize animal suffering.

Preparation of Wild Bitter Gourd Powder

Wild bitter gourd cultivars (MC2, 3, 5, and 5523) were obtained from the Hualien District Agricultural Research and Extension Station, Council of Agriculture, Executive Yuan, Taiwan. Whole fruit was cut into small pieces. The pieces were collected, freeze-dried and finely ground. The freeze-dried powders were extracted with 80% ethanol (1:20 *w*/*v*). Then, the solutions were centrifuged at 4500× *g* for 30 min and filtered. The filtrates were concentrated by a rotary evaporator to remove the ethanol. The concentrates were freeze-dried to remove the remaining water. Finally, the dried ethanol extracts of MC (18.7% for MC2, 12.9% for MC3, 9.55% for MC5, and 12.15% for MC5523) were obtained and dissolved in DMSO (0.1%) for use in vitro.

Immunocytochemistry Analysis

Cells (n = 3–5 cultures per group, 3 wells/culture) were harvested for immunocytochemical (ICC) staining after the different treatments for 24 h in 10 mM glucose or 1 h in 10 nM WT/GFX. The cells were first fixed with ice-cold 4% paraformaldehyde (Sigma-Aldrich) for 30 min and washed with phosphate-buffered saline with Triton X-100 (PBST) 3 times for 10 min each. Non-specific reactivity was blocked by incubation with 10% fetal bovine serum (FBS) for 2 h. Cells were then incubated with NeuN (1:1000; Millipore) and MAP2 (1:1000; Millipore) primary antibodies for 16 h at 4 °C, followed by incubation with a secondary antibody for 2 h at 37 °C. Finally, the neuronal nuclei were counterstained with 4′,6-diamino-2-phenylindole (DAPI; Sigma-Aldrich) and immediately analyzed using a High Content Micro-Imaging Acquisition and Screening System (Molecular Devices, Sunnyvale, CA, USA). Both the numbers of neurons and neurite length were analyzed using MetaXpress application software (Molecular Devices).

LDH Assay

The LDH assay was used to measure cell death in vitro (n = 3–5 cultures per treatment, 3 wells/culture). LDH is a stable cytoplasmic enzyme present in all cells that is rapidly released into the culture medium upon damage of the plasma membrane. Therefore, LDH leakage into the medium is a marker of cell damage. The primary hippocampal neuronal culture supernatants were

collected after the different treatments and quantified the LDH leakage using an LDH Cytotoxic Test kit (Clontech) according to the manufacturer's instruction.

Supplemental Results

The Neuroprotective Effects of MC5, MC3, MC2, and MC5523 in Mouse Primary Hippocampal Neurons under Hyperglycemia

The results of screening 4 different MC varieties and LiCl showed that the neuronal percentage was significantly increased for MC2, 3, and 5 at 1, 10, 50, and 100 μg/mL ($p < 0.001$; Figures S1B and C); MC5523 at 1 μg/mL ($p < 0.001$; Figures S1B and C) 10, 50, and 100 μg/mL ($p < 0.01$; Figures S1B and C); and LiCl at 2.5, 5 mM ($p < 0.01$; Figures S1B and C) 10, and 50 mM ($p < 0.001$; Figures S1B and C) against damage from 10 mM glucose in the mouse primary hippocampal neuronal culture. The neurite length analysis showed that the neurite length was significantly increased for MC5 at all doses ($p < 0.001$; Figures S1B and D); MC3 at 1 μg/mL ($p < 0.01$, Figures S1B and D), 10, 50, and 100 μg/mL ($p < 0.001$; Figures S1B and D); MC2 at 1, 50 μg/mL ($p < 0.01$), 10, and 100 μg/mL ($p < 0.05$; Figures S1B and D); MC5523 at 1, 10 μg/mL ($p < 0.01$), 50, and 100 μg/mL ($p < 0.001$; Figures S1B and D); and LiCl at 2.5, 5.0 mM ($p < 0.001$), and 10 mM ($p < 0.01$; Figures S1B and D). The branch analysis showed that the relative percentage of branch number was significantly increased for MC3, and MC5 at all doses ($p < 0.001$; Figures S1B and E) and MC2 at 1, 10, 50 μg/mL ($p < 0.001$), and 100 μg/mL ($p < 0.05$; Figures S1B and E) but decreased for LiCl at 50 mM ($p < 0.05$; Figures S1B and E). The above results suggest that all the four MCs have neuroprotective effects in mouse primary hippocampal neurons incubated with 10 mM glucose. In addition, high-dose (50 mM) LiCl treatment induced neurite damage. Therefore, both MC5 (1μg/mL) and MC5523 (10 μg/mL) were selected for studying the effects of combination with LiCl (5 mM).

Neuroprotective Effect of LiCl and MC5523 Cotreatment Was Superior to that of LiCl and MC5 in Primary Hippocampal Neurons Treated with WT/GFX

To determine the combined neuroprotective effect of LiCl (5 mM) plus MC5 (1 μg/mL) or MC5523 (10 μg/mL), the treatments were applied to mouse primary hippocampal neurons on DIV 9 after treatment with 10 nM WT/GFX for 1 h. We found that the neuroprotective effect of LiCl plus MC5523 ($p < 0.01$–0.001) was better than that of LiCl with MC5 ($p < 0.01$; Figure S2).

Supplemental Figures

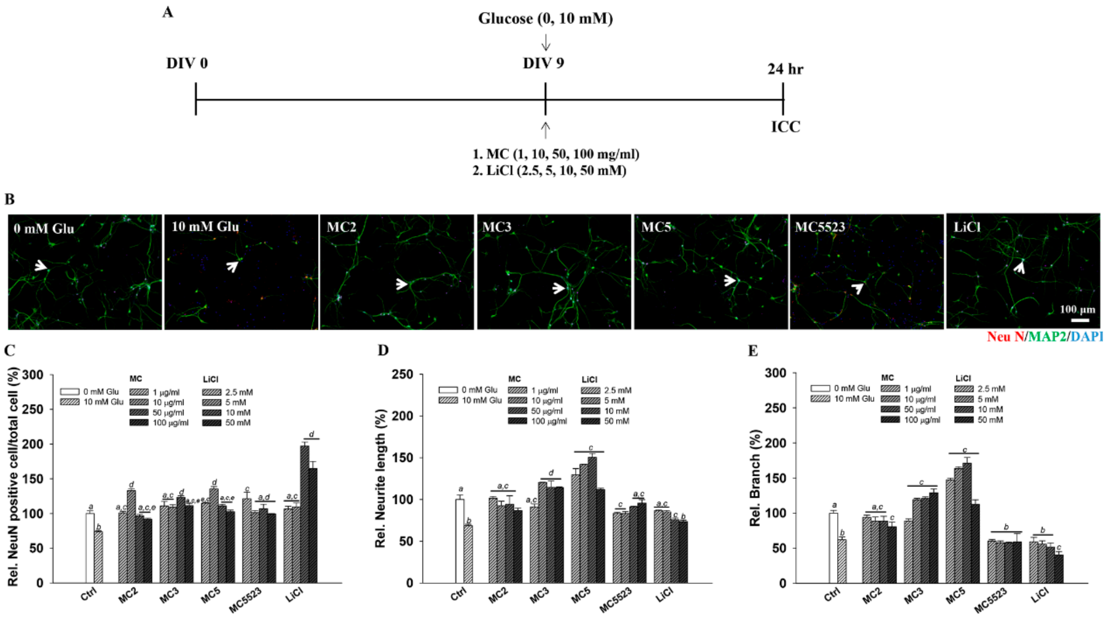

Figure S1. The different dosage effects of the four strains of MC and LiCl on mouse hippocampal primary neurons cotreated with 10 mM glucose. **(A)** The timeline of MC and LiCl treatment with different doses applied to mouse primary hippocampal neurons cotreated with 10 mM glucose. **(B)** The immunostaining results of mature neurons and neurite length and branching after cotreatment with 10 mM glucose, MC, and LiCl. **(C–E)** Quantification of the mature neurons, the neurite length and the neurite branch number relative to all cells in primary cultures treated with 10 mM glucose, MC, and LiCl (*n* = 3–5 cultures per group, 3 wells/culture). Data are expressed as the mean ± SEM. Means that do not share a letter are significantly different ($p < 0.05$).

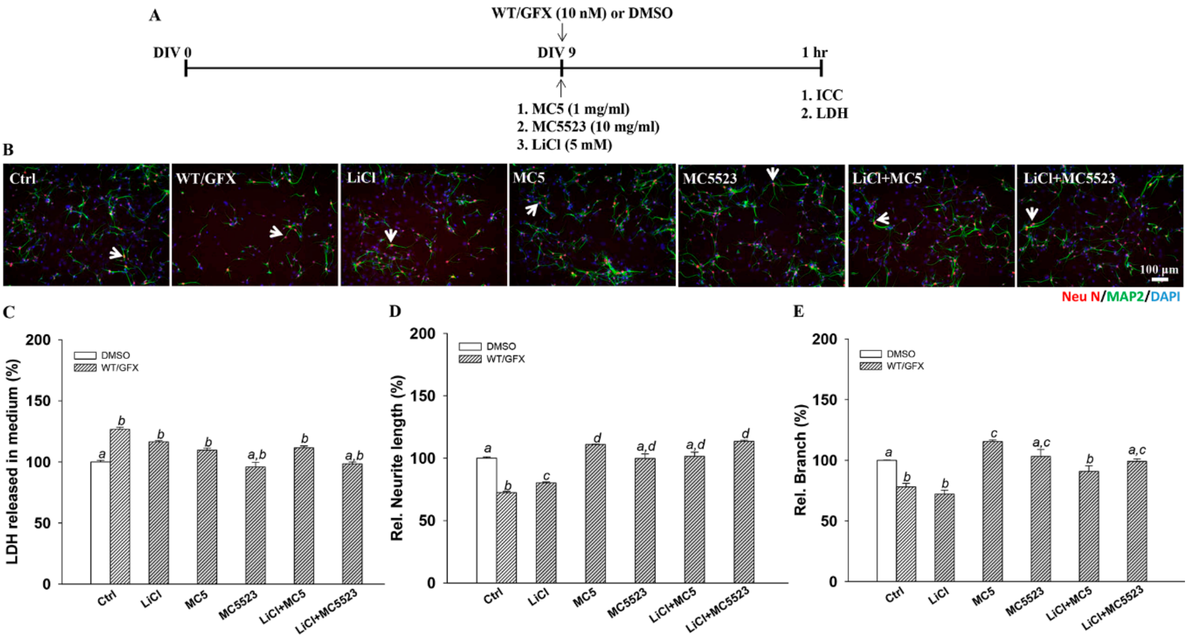

Figure S2. The combined effects of MC5 or MC5523 and LiCl on mouse hippocampal primary neurons cotreated with 10 nM WT/GFX. **(A)** The timeline of mouse primary hippocampal neuron treatment with MC5, MC5523 and/or LiCl under 10 nM WT/GFX conditions. **(B)** The immunostaining results of mature neurons and neurite length and branching after cotreatment with WT/GFX, MC, and LiCl. **(C–E)** Quantification of the cellular death rate and the neurite length and branch number relative to all cells cotreated with WT/GFX, MC, and LiCl (*n* = 3–5 cultures per group, 3 wells/culture). Data are expressed as the mean ± SEM. Means that do not share a letter are significantly different ($p < 0.05$).

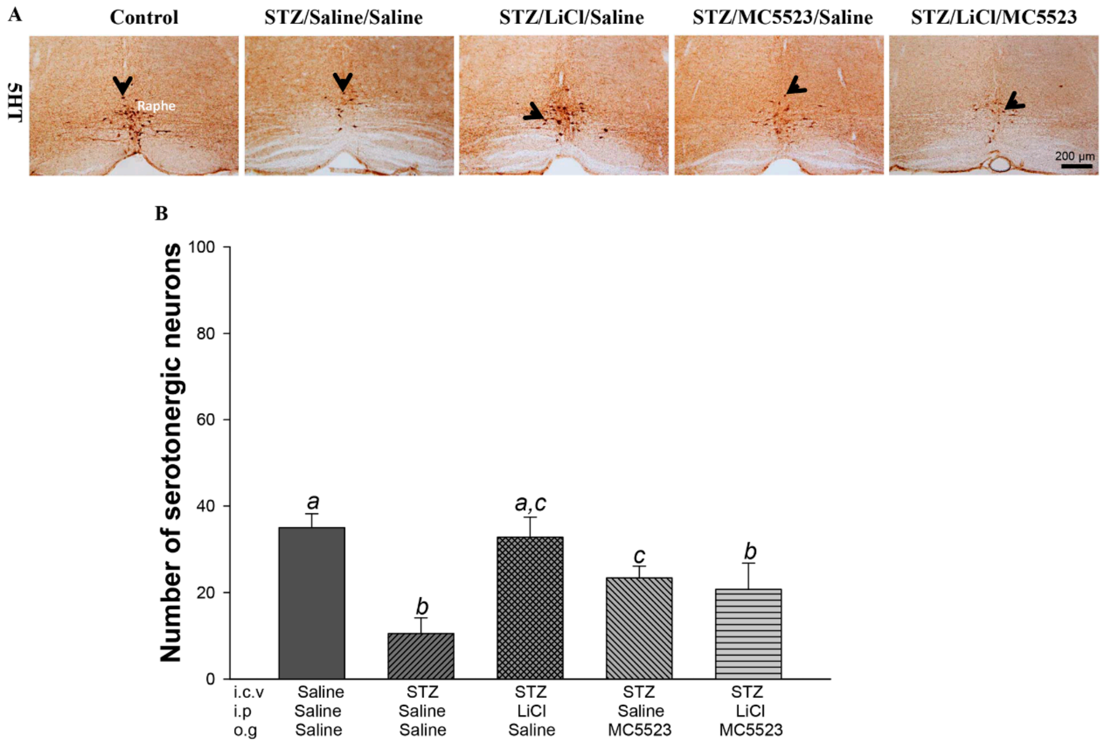

Figure S3. The cotreatment of LiCl and MC5523 had no effect on the serotonergic neuronal loss in icv-STZ B6 mice. **(A)** Representative images of serotonergic neurons (with 5-HT staining) in the Raphe nucleus. Scale bar = 200 μm, and arrowheads indicate positive staining signals. **(B)** The number of serotonergic neurons. The results show that the serotonergic neurons were increased in number in the group treated with LiCl or MC5523 alone, but not in the group treated with LiCl plus MC5523. The quantitative data are shown as the mean ± SEM of each group (n = 3 per group). Means that do not share a letter are significantly different ($p < 0.05$).

Supplemental Table

Table S1. Exterior characteristics of MC5523.

Characterization / Variety	Fruit Weight (g)	Fruit Length (mm)	Fruit Width (mm)	Peduncle Length (mm)	Fruit Thickness (mm)
MC5523 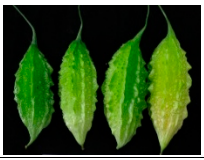	45.10 ± 5.72	100.47 ± 4.45	34.68 ± 1.95	70.51 ± 5.68	4.96 ± 1.24
MC5 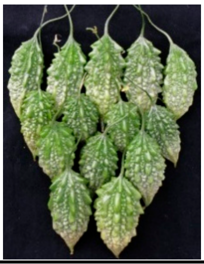	28.30 ± 7.70	73.50 ± 0.10	33.40 ± 3.50	84.50 ± 29.81	11.40 ± 0.90

Supplemental References

1. Seibenhener, M.L.; Wooten, M.W. Isolation and culture of hippocampal neurons from prenatal mice. *J. Vis. Exp.* **2012**; DOI: 10.3791/3634.
